# The Relationship between Duration of Smartphone Uses and Anxiety in University Students during the COVID-19 Outbreak

**DOI:** 10.3390/ijerph19116620

**Published:** 2022-05-29

**Authors:** Jianmin Wang, Wang Li, Liang Ding, Shulei Chen

**Affiliations:** 1 Department of Physical Education, Huaiyin Institute of Technology, Huaian 223003, China; liwang0518@hyit.edu.cn; 2 Department of Physical Education, Southeast University, Nanjing 211189, China; 101011543@seu.edu.cn; 3 Department of Physical Education, Dalian University of Science and Technology, Dalian 116052, China; chen_shulei@hotmail.com

**Keywords:** smartphone usage, anxiety, university students, COVID-19

## Abstract

**Background:** During the COVID-19 pandemic, China adopted a home isolation policy, which caused lifestyle changes for university students, including increased smartphone use. Several studies indicate that problematic smartphone use is associated with anxiety. However, this association has not been examined in the context of epidemics. The aim of this study was to investigate whether the duration of smartphone use was associated with anxiety in Chinese university students during the COVID-19 pandemic. **Methods:** Participants included 9716 university students (5458 men and 4258 women) from Liaoning, China. We assessed the duration of smartphone use with a self-reported questionnaire. Anxiety was assessed using the generalized anxiety disorder seven-item scale. A multivariate logistic regression analysis was performed to determine the adjusted association between smartphone use and anxiety. **Results:** After adjusting for confounding factors, we observed a positive association between smartphone use duration and the prevalence of anxiety in all participating students. Compared with short periods of smartphone usage, the odds ratios (95% confidence interval) for moderate and long smartphone usage duration were 1.17 (1.00, 1.36) and 1.58 (1.36, 1.83), respectively. This significant positive association did not change in the sex-stratified analysis (for both men and women). **Conclusions:** Our examination of the association between duration of smartphone uses and university students’ anxiety levels revealed that long smartphone use was associated with a high prevalence of anxiety during the COVID-19 pandemic.

## 1. Introduction

Anxiety disorders are among the most common psychiatric disorders [1] and are always associated with negative health outcomes. Reports associate anxiety with poor physical and medical conditions, such as chronic diseases and diabetes [2]. In addition, individuals with anxiety have a higher risk for hypertension than people without anxiety [3]. High levels of anxiety have been reported by university students [4]. Young adults with mental illnesses usually experience impaired self-esteem, bullying, and failed family and social relationships [5]. Young adulthood is an important period that links adolescence with mature adulthood. Thus, ensuring good mental health in this period is important, as it may have a lifetime effect.

In modern daily life, technology occupies an extremely important position. Smartphones are high-tech products that impact people’s lives. The number of global smartphone users reached 3.5 billion in 2020, an increase of 9.3% since 2019 [6]. Compared with traditional mobile phones—used only to make and receive calls—smartphones have additional functions, allowing users to conveniently browse the internet, listen to music, shop, and send emails. On the other hand, improper use of smartphones can produce many negative effects. For example, previous studies point to associations between smartphone use and muscle pain, fatigue [7], and upper limb musculoskeletal disorders [8]. Participants in previous studies who had smartphone addictions engaged in less physical activity, resulting in an increase in fat mass [9]. Based on these findings, we considered that smartphone usage could also be associated with anxiety, because low levels of physical activity and obesity are risk factors for poor mental health.

Several studies have explored the association between smartphone use and anxiety. However, some were focused on specific populations (not young adults), and some used the anxiety score as a continuous variable or did not treat anxiety as a symptom. At the end of 2019, COVID-19 was rapidly spreading across China, with more than 80,000 confirmed cases in March 2020 [10]. Consequently, China’s home isolation policy altered people’s living conditions significantly. For example, reduced activity space led to a decrease in physical activity. Students began using mobile applications to complete their homework. Increased time spent on smartphones for various reasons might have had a negative impact, such as anxiety. However, no research has been conducted to demonstrate the relationship between smartphone use and anxiety during the COVID-19 pandemic. Therefore, we designed a cross-sectional study to investigate the association between smartphone use and anxiety experienced by university students during the pandemic. We hypothesized that longer smartphone usage is associated with a high risk for anxiety.

## 2. Materials and Methods

### 2.1. Sample and Procedures

The study participants were 11,000 university students enrolled in two universities in Liaoning, China. Of these, 6200 (56.4%) were men and 4800 (43.6%) were women. The institutional review board of the Dalian University of Science and Technology approved all testing procedures. Because of home quarantine, we recruited participants from announcements made via cellphone massages sent to student groups. We requested that students complete our survey using a mobile application that explained study details and the procedure for granting informed consent. We collected data between June and July 2020 and performed the analysis beginning in March 2021. Responses from 1284 participants were excluded because of missing data. Therefore, the final number of participants was 9716 (men 5458, women 4258).

### 2.2. Smartphone Usage

We measured smartphone use duration with the question, “How many hours on average did you use your mobile phone every day?” Respondents replied with “___hours, ___minutes.” Subsequently, we obtained the total minutes of smartphone usage for the analysis.

### 2.3. Anxiety Symptoms

The generalized anxiety disorder seven-item scale (GAD-7) was used to assess anxiety based on self-reported measures. This tool is useful for screening anxiety symptoms. The GAD-7 consists of seven items that assess general anxiety symptoms mentioned in the *Diagnostic and Statistical Manual of Mental Disorders, 4th edition* (DSM-IV). Participants rated their anxiety-related problems for the previous two weeks on a four-point scale (from 0 = not at all to 3 = almost every day). The total scores ranged from 0 to 21; higher scores indicated an increased severity of symptoms. The GAD-7 is regarded as a reliable tool for anxiety assessment [11]. In the present study, we used a cutoff value of 10 to define anxiety symptoms [12].

### 2.4. Covariates

Students provided information requested via the questionnaire developed for this study regarding sex (i.e., male or female), grade (i.e., freshman, sophomore, senior, or junior), race (i.e., Han nationality or minority race), family relationships (i.e., good or other), living situation (i.e., lives with parents or other), smoking status (i.e., smoker or nonsmoker), drinking status (i.e., nondrinker, drinks occasionally, or drinks every day), and sleep quality (i.e., good or poor). Family income categories were ≤ CNY 5000 (low income), CNY 5001–12,000 (middle income), and > CNY 12,000 (high income). Levels of physical activity (PA) were assessed using the International Physical Activity Questionnaire (IPAQ) [13]. Total PA was measured with metabolic equivalents [METs] in hours/week and subsequently categorized as low, medium, or high.

### 2.5. Statistical Analysis

We performed a multivariate logistic regression analysis to examine the relationship between smartphone use and anxiety. Descriptive data were presented as odds (with 95% confidence intervals [95% CIs]). Anxiety was a dependent variable, whereas categories of smartphone usage (in tertiles) were independent variables. Significance of differences within categories of smartphone use duration was assessed using the Bonferroni test. We conducted an analysis of covariance to study the correlation between smartphone usage and anxiety in the unadjusted and adjusted models. Model 1 incorporated adjustments for grade, sex, and race, and Model 2 incorporated adjustments for grade, sex, race, family income, family relationships, living conditions, tobacco smoking, drinking status, physical activity, and sleep quality. We performed a statistical analysis using SPSS Statistics version 24.0 for Windows (SPSS, Inc., Chicago, IL, USA) and analyzed all p-values in the unadjusted model, Model 1, and Model 2 for trends. A *p*-value of less than 0.05 was considered statistically significant.

## 3. Results

All students who participated in this study used smartphones. Of the 9716 participants, 5458 (56.2%) were men; 1165 (12.0%) students reported having anxiety symptoms. Table 1 shows participants’ characteristics according to their anxiety levels. Freshmen had a greater prevalence of anxiety. The percentage of participants classified as seniors who reported having good relationships with their families, as well as good sleep quality, were likely to have lower levels of anxiety.

As shown in Table 2, the multivariate logistic regression analysis revealed the relationship between smartphone usage and anxiety in the study participants. The group with short periods of smartphone usage was the reference group. Study findings indicated a significant inverse association between mobile phone usage and anxiety. We compared the unadjusted model to the reference group, and the odds and 95% CIs of moderate and long smartphone usage categories were 1.17 (1.00, 1.36) and 1.58 (1.36, 1.83), respectively (*p* < 0.001). This strong significant association did not change after adjustment for confounding factors in Models 1 and 2. The prevalence of anxiety was higher for the long duration of smartphone use category than for the short category in final adjusted model (Model 2).

Table 3 presents the adjusted association between mobile phone usage and anxiety in men and women. In a comparison with short smartphone usage, the odds ratios (95% CIs) of the unadjusted model for men for moderate and long smartphone usage were 1.27 (1.04, 1.56) and 1.83 (1.50, 2.24), respectively. This significant association did not change in the adjusted Models 1 and 2. Regarding the same comparison for the unadjusted model for women, the odds (95% CIs) for moderate and long smartphone usage were 1.01 (0.80, 1.27) and 1.28 (1.03, 1.60), respectively (*p* = 0.024). This association was stronger after making adjustment in Models 1 and 2 (*p* = 0.003 and 0.008, respectively). The prevalence of anxiety was higher for the moderate and the long duration of smartphone use categories than for the short category for men, and was higher for the long duration of smartphone use category than for the short category for women in the final adjusted model (Model 2).

## 4. Discussion

In this cross-sectional study, we established an independent association between smartphone usage and anxiety in Chinese university students. Results revealed that a longer duration of smartphone usage is associated with a higher prevalence of anxiety. Subgroup analyses also confirmed the same association for both men and women. Our findings not only strengthened the evidence of the association between smartphone usage and anxiety, but also initially verified this association during the COVID-19 pandemic.

The prevalence of anxiety (GAD-7 score ≥ 10) in our study was 12.0%, a figure consistent with findings of a previous study that showed an 11.9% prevalence of anxiety in Saudi university students during the COVID-19 pandemic [14]. However, the prevalence of anxiety found in our study was lower than that in a Swiss study and a Polish study, which reported anxiety levels at 16.0% and 35.0%, respectively, among university students during the pandemic [15,16]. Another study reported that the prevalence of anxiety experienced by Chinese university students in COVID-19 was 6.35% [17], which was lower than that found in our study.

With the increased use of smartphones in recent years, overuse has also escalated, along with the potential for various psychological problems. The relationship between smartphone usage and anxiety has been evaluated in other studies. Miles et al. reported that problematic smartphone use (PSU) was positively related to anxiety in 244 British adults [18]. Although, their findings were partially consistent with this study’s, the participants were adults (mean age 29.7 years) but not university students. On the other hand, Kadir et al. found that anxiety scores were higher for the group with high levels of smartphone use than for the group with low levels of smartphone use in a sample of 319 Turkish university students [19]. Lee et al. found that, among 1236 university students in South Korea, smartphone dependency appeared to be associated with increased anxiety [20]. The results of another study indicated that addiction to mobile phones led to anxiety in a group of 785 Italian college students [21]. The findings of these previous studies corresponded to those of our study; likewise, we all examined the relationship between smartphone use and anxiety in university students. However, the assessments employed to measure smartphone use and anxiety were different. We used the GAD-7 to evaluate anxiety and a cutoff value to define participants with or without anxiety; we did not treat anxiety as continuous variable. Furthermore, our sample size was larger than those in the other studies. We considered mental health to be different in men and women, so we performed subgroup analyses by sex, and we found that although long duration of smartphone use is associated with higher risk of anxiety in both men and women, there is a difference that exists between the genders. In addition, this study was the first to investigate the relationship between smartphone use and anxiety in Chinese university students during the COVID-19 pandemic. An Australian study revealed that communicative smartphone use could decrease anxiety by increasing friendship satisfaction [22]. This finding is not consistent with the present study. The primary difference between these studies is that the present study did not classify smartphone use by purpose (communicative or non-communicative). Furthermore, the present study assessed smartphone use by duration, not frequency. These differences may cause inconsistencies between the studies. It is possible that, although longer smartphone use is associated with a higher prevalence of anxiety, the communicative purposes of smartphone use have a positive effect on reducing anxiety. This possibility needs to be investigated in future studies.

Although the exact mechanisms are not clear, there are some possible explanations for the association between smartphone usage and anxiety. First, it is well known that sleep and eating behaviors affect mental health [23,24]. To ensure a uniform schedule, the vast majority of Chinese university students live in dormitories. However, during the COVID-19 pandemic, students quarantined at home. Without a managed schedule, they often used smartphones longer than they should have at night, which might have caused changes in sleep patterns, such as falling asleep later or not getting enough sleep. Such changes in sleep patterns inevitably lead to problematic eating behaviors, such as skipping breakfast or eating late. Second, an increase in smartphone use is associated with a reduction in physical activity [25], which is also a potential factor for anxiety. Third, a previous study indicated that exposure to mobile phone emissions at nighttime could affect melatonin onset time [26]. Melatonin is effective in reducing anxiety [27]. Finally, smartphone overuse is associated with an increase in headache intensity and duration [28], which may also influence anxiety.

Several limitations of this study should be considered. First, the cross-sectional design of the study prevented causal inference regarding whether longer duration of smartphone use contributed to increased prevalence of anxiety or students experiencing anxiety tended to longer duration of smartphone use. This is a cross-sectional study; thus, no clear cause–effect conclusion could be directly drawn. Second, the data about confounding factors were limited. We cannot exclude the possibility that other covariates might affect the association between smartphone usage and anxiety, such as dietary behaviors and health status. Third, the participants in this study were from only one area in China, pointing to a section bias. Additionally, our sample of university students with advanced educational levels does not fully represent the general Chinese population. Studies of other populations should be conducted. Fourth, respondents reported smartphone usage and anxiety, although most smartphones can automatically remind users of their weekly usage amounts. Furthermore, since the GAD-7 is a validated questionnaire, a recall bias may also exist. Fifth, we collected the data during the COVID-19 pandemic; thus, the study findings may only be representative of a special period. Finally, we did not obtain data regarding family histories of anxiety or other diseases that could affect the findings.

## 5. Conclusions

Despite the stated limitations, this study contributes to an understanding of the relationship between smartphone use and anxiety among Chinese university students during the COVID-19 outbreak. The results showed that lengthy smartphone usage duration was significantly related to a high prevalence of anxiety, and this association was independent with confounding factors. Although this relationship was revealed during the COVID-19 outbreak—a distinct period in modern history—it could be important information applicable to similar future situations. Considering that smartphone usage is playing an important role in university students’ daily life, this association may also apply to normal periods when the COVID-19 quarantine is over. Furthermore, the results provide vital information for the preventive medicine field. According to these results, it is necessary to control the use of smartphones in the case of epidemic emergencies, which relates to the effective prevention of anxiety disorders to a certain extent in university students. In the future, a randomized controlled study or prospective study should be conducted to clarify the causality.

## Figures and Tables

**Table 1:** Participant characteristics according to anxiety.

	Anxiety		*p* Value ^a^
	No	Yes	
*n*	8551	1165	
Sex (men; %)	56.5	54	0.116
Minority race (%)	4.7	5.5	0.242
Grade (%)			
Freshman	26.2	44.7	<0.001
Sophomore	28.0	24.5	
Junior	26.9	22.7	
Senior	18.9	8.2	
Physical activity (%)			
Low	33.3	33.1	0.981
Medium	33.2	33.5	
High	33.5	33.4	
Family income (%)			
Low	37.8	41.2	0.083
Medium	52.1	49.4	
High	10.1	9.4	
Family relationships (good; %)	88.6	75.9	<0.001
Living condition (with parents; %)	92.1	90.5	0.059
Smoking status (%)			
Smoker	5.3	5.8	0.405
Drinking status (%)			
Nondrinker	80.4	77.8	0.116
Drink occasionally	17.7	19.9	
Drinking everyday	2.0	2.3	
Sleep quality (good; %)	82.0	62.0	<0.001

^a^ Obtained using *X*^2^ test for proportional variables.

**Table 2:** Adjusted relationship between smartphone use duration and anxiety in all participants ^a^.

	Smartphone Use Duration			*p* Value ^b^
	Short	Moderate	Long	
*n*	3716	3148	2852	
Anxiety (GAD score ≥ 10); *n*	373	363	429	
Unadjusted model	1	1.17 (1.00, 1.36) *	1.58 (1.36, 1.83) *	<0.001
Model ^c^	1	1.17 (1.00, 1.36) *	1.64 (1.41, 1.91) *	<0.001
Model ^d^	1	1.15 (0.98, 1.35)	1.62 (1.39, 1.89) *	<0.001

^a^ Variables are expressed as odds (95% CIs). ^b^ Obtained using multivariate logistic regression, *p* value is for trend. ^c^ Adjusted for sex, grade, and race. ^d^ Adjusted for sex, grade, race, family income, family relations, living condition, tobacco smoking, alcohol drinking, physical activity, and sleep quality. * Significantly different to short category, *p* < 0.05.

**Table 3:** Sex-stratified analysis for the relationship between smartphone use duration and anxiety ^a^.

	Smartphone Use Duration			*p* Value ^b^
	Short	Moderate	Long	
Men; *n*	2407	1661	1390	
Anxiety (GAD score ≥ 10); *n*	221	190	218	
Unadjusted model	1	1.27 (1.04, 1.56) *	1.83 (1.50, 2.24) *	<0.001
Model 1 ^c^	1	1.26 (1.02, 1.55) *	1.85 (1.51, 2.27) *	<0.001
Model 2 ^d^	1	1.24 (1.01, 1.54) *	1.85 (1.50, 2.27) *	<0.001
Women; *n*	1309	1487	1462	
Anxiety (GAD score ≥ 10); *n*	152	173	211	
Unadjusted model	1	1.01 (0.80, 1.27)	1.28 (1.03, 1.60) *	0.024
Model 1 ^c^	1	1.04 (0.82, 1.32)	1.40 (1.11, 1.75) *	0.003
Model 2 ^d^	1	1.01 (0.80, 1.28)	1.35 (1.07, 1.70) *	0.008

^a^ Variables are expressed as odds (95% CIs). ^b^ Obtained using multivariate logistic regression, *p* value is for trend. ^c^ Adjustment for grade, and race. ^d^ Adjustment for grade, race, family income, family relations, living condition, tobacco smoking, alcohol drinking, physical activity, and sleep quality. * Significantly different to short category, *p* < 0.05.

## Data Availability

The data presented in this study are available on request from the corresponding author.
